# Therapeutic opportunities of fecal microbiota transplantation in the treatment of non-alcoholic fatty liver disease

**DOI:** 10.3389/fcimb.2026.1962509

**Published:** 2026-10-28

**Authors:** Ying Chen, Yu Li, Xiaojuan Li, Xinxin Xu, Shiyao Chen

**Affiliations:** 1 Department of Gastroenterology and Hepatology, Minhang Hospital, Fudan University, Shanghai, China; 2 Department of Gastroenterology and Hepatology, Zhongshan Hospital, Fudan University, Shanghai, China

**Keywords:** clinical benefit, engraftment, fatty liver disease, fecal microbiota transplantation, gut microbiota

## Abstract

Based on the emerging evidence in the crosstalk between gut microbiota composition and the non-alcoholic fatty liver disease (NAFLD), the application of fecal microbiota transplantation (FMT) on patients with NAFLD has become an attractive research field. Evidence from multiple studies have proved that lean patients with NAFLD were more sensitive to FMT in both microbiomic and metabolic aspects compared to obese patients, and more benefits were observed in patients receiving allogenic FMT from lean donors compared to those receiving autologous FMT, possibly due to the benefits derived from the successful engraftment of donor’s microbiota post FMT. However, it has been observed from the clinical practice that an identified successful engraftment is not a promise of clinical efficacy in NAFLD patients. This review aims to summarize the current evidence supporting application of FMT in treatment of NAFLD. We realized that the clinical efficacy and long-term benefits of FMT on NAFLD patients warrant further investigation with more properly-designed randomized controlled trials.

## 1 Introduction

Characterized by having more than 5% fat by volume in the liver, metabolic dysfunction-associated fatty liver disease (MAFLD), formerly named non-alcoholic fatty liver disease (NAFLD), is a spectrum of liver disease ranging from isolated steatosis to non-alcoholic steatohepatitis (NASH) (Eslam et al., 2020). Metabolic dysfunction-associated steatotic liver disease (MASLD) is another term that covers a broader conditions, with an alcohol-related overlap and a higher number of lean individuals (body mass index (BMI)<25kg/m^2^) included. Researchers have stated that NAFLD may become the most common indication for liver transplantation by 2030 (Danford and Lai, 2019). NAFLD is estimated to affect approximately a quarter of the general adult population around the world, hence posing a heavy health and economic burden to all societies (Younossi et al., 2018; Riazi et al., 2022). The prevalence climbs towards a significantly higher level of 55% in individuals with type 2 diabetes mellitus (T2DM) (Younossi et al., 2019a; Huang et al., 2021), and to over 90% in those with obesity (BMI≥30kg/m^2^) (Younossi et al., 2019b; Quek et al., 2023). Due to a lack of proven or registered pharmacotherapy to reduce the disease burden, there is an urgent need to develop novel therapeutic targets for NAFLD. The alterations in gut microbial composition have been continuously implicated in the pathology of fatty liver disease with different severity (Boursier et al., 2016; Loomba et al., 2019), via different mechanisms such as modulation of bile acids (Puri et al., 2018). Therefore, a variety of approaches modulating an individual’s gut microbiota have been attempted to test their potentials in treatment of NAFLD, with fecal microbiota transplantation (FMT) being one of these approaches.

As a procedure in which feces of a healthy donor (allogenic) or an individual’s own feces (autologous) is transplanted into the gastrointestinal tract of a recipient, FMT has become an increasingly utilized approach to treat a wide range of diseases by modulating gut microbiota compositions (Peery et al., 2024). Based on the current guidelines and consensus (Cammarota et al., 2019), healthy donors are required to pass medical interview as well as blood and stool testing to exclude existence of Clostridioides difficile, common enteric pathogens, antibiotic-resistant bacteria, norovirus, rotavirus, adenovirus, *Giardia lamblia*, *Cryptosporidium spp*, *Isospora* and microsporidia, *Protozoa* and helminths/va and parasites, as well as *Helicobacter pulori* fecal antigen if FMT delivery via upper route is adopted. To date, FMT has shown certain promising efficacy in improvement and treatment of infection (*Clostridioides difficile*) (Surawicz et al., 2013), inflammatory bowel diseases (Crohn’s disease and ulcerative colitis) (Kao et al., 2024; Zhang et al., 2024a; Lu et al., 2025) and metabolic syndrome (Ng et al., 2022; Wortelboer et al., 2023; Zhang et al., 2024b). However, research on application of FMT to NAFLD patients is still scarce, and the separation of ‘a successful transplantation’ and ‘beneficial clinical outcomes’ is not yet fully understood. Previous reviews have evaluated microbial signatures with different stages of diseases (Aron-Wisnewsky et al., 2020), synthesized pathogenic mechanisms with newly emerged therapeutics (Schnabl et al., 2025) or integrated gut microbiota, immunometabolism and gut-liver axis (Tilg et al., 2022) with a multi-omics focus. However, there is lack of summarization integrating machine-learning to establish models for various needs, nutrition and precision-medicine with patient stratification in NAFLD. An understanding and consideration of these aspects will provide valuable information to decisions in strategy making in clinical practice.

This review aims to summarize current evidence supporting application of FMT in treatment of NAFLD, specifically by covering several genuinely important questions, including (1) different FMT responsiveness when FMT recipients or donors were lean, particularly focusing on the existing microbiome and metabolic alterations that may be correlated with this heterogeneity; (2) impact of the microbiome shifts from FMT on the gut of NAFLD patients; (3) functional metabolic consequences derived from FMT that brings benefits to NAFLD; (4) potential reason for engraftment-vs-clinical-benefit dissociation; and (5) limitations in current studies.

## 2 Methods

This narrative review includes a database search of published articles following a pre-defined searching strategy. Records were searched through PubMed, Scopus, Embase, Web of Science Core Collection and Cochrane CENTRAL, using the search term (‘fecal microbiota transplantation’ OR ‘faecal microbiota transplantation’ OR ‘FMT’) AND (‘Non-alcoholic fatty liver disease’ OR ‘NAFLD’ OR ‘metabolic dysfunction-associated steatotic liver disease’ OR ‘MASLD’ OR ‘metabolic dysfunction-associated fatty liver disease’ OR ‘MAFLD’ OR ‘metabolic dysfunction-associated steatohepatitis’ OR ‘MASH’ OR ‘non-alcoholic steatohepatitis’ OR ‘NASH’ OR ‘fatty liver disease’ OR ‘hepatic steatosis’) for publications in English within the last ten years until 08 January 2026. A total of 1465 English articles were yielded for subsequent screening. Then titles and abstracts of these articles were further processed by two independent authors for detailed review. Once discrepancies occur, they were evaluated by the corresponding author. Relevant information was extracted from articles in these searches and selections. We also conducted a grey literature search through the Google Scholar search engine platform to identify the relevant studies that is not captured through database search. Studies evaluating application of FMT in NAFLD or related terms were included.

## 3 FMT responsiveness when recipients or donors were lean

NAFLD may occur in both obese and lean populations (Consultation, W. H. O, 2004). Evidence from multiple published studies have all proved that NAFLD patients who were lean or received FMT from lean donors were more sensitive to this treatment from both metabolic and microbiomic aspects compared to their comparators (**Table 1**; **Figure 1**). In addition, more evidence may come out later with publication of results from registered clinical studies (**Table 2**).

**Table 1:** Randomized controlled trials (RCTs) on the application of FMT in the treatment of patients with NAFLD.

Ref	FMT recipients (R) and donors (D)	Groups	FMT frequency, duration, route	Detection of microbiome	Direct outcomes of liver (data if available)	Other outcomes (data if available)	Performance (AUC)	Conclusion
(Witjes et al., 2020; Stols-Goncalves et al., 2023)	R: NAFLD patients D: lean vegan donors	• Group 1: allogenic FMT (n=10) • Group 2: autologous FMT (n=11)	3 times in 8 weeks, duodenal infusion	• multi-omics machine learning approach	• hepatic DNA methylation profiles from paired liver biopsies before/after FMT • Fibrosis score# (allogenic: 1.2 ± 0.92 vs 1.60 ± 0.70; autologous: 0.91 ± 0.70 vs 1.18 ± 0.75)	• gut microbiota profiles • plasma metabolites	Gut microbiota: 0.78 Plasma metabolomics: 0.74 Liver DNA methylation profiles: 0.75	In group 1 compared to group 2: • ↑Eubacterium siraeum, Blautia wexlerae • PAC, PAG • TARS, ZFP57
(Groenewegen et al., 2025)	R: Obese adult patients with MASLD D: two lean donors	• Group 1: allogenic FMT (n=10) • Group 2: autologous FMT (n=10)	3 times at week 0, 3 & 6, gastroduodenal endoscopy	• metagenomic sequencing	• MRI-PDFF for hepatic steatosis $ (0.13 [0.06-0.19], p=0.002) • liver biochemistry	• glucose tolerance • Insulin resistance$ (-0.03 [-0.07-0.01]) • gut microbiota composition/engraftment	Glucose: -9.64 Insulin: -33.61 C-peptide: 13.06 GIP: -7.33 GLP-1: -127.32	In group 1 compared to group 2: • (-) MRI-PDFF, liver biochemistry, glucose tolerance from baseline to week 12 • Gastranaerophilaceae and Rikenellaceae families were associated with triglyceride levels after FMT.
(Xue et al., 2022)	R: NAFLD patients both obese and lean D: healthy young donors	• Group 1: allogenic FMT (n=28) • Group 2: oral probiotics^*^ (n=47)	3 times in 3 days, first colonscopy and then three enemas	• 16S rRNA	• fatty liver severity • fatty deposition in the liver (Lean#: 253.4 ± 36.2 vs 235.7 ± 24.5; Obese#: 285.2 ± 42.3 vs 279.5 ± 44.5)	• blood lipid, blood glucose, blood pressure	/	In lean patients compared to obese patients: • higher clinical efficacy after FMT
(Craven et al., 2020)	R: NAFLD with metabolic syndrome D: three healthy lean donors	• Group 1: allogenic FMT (n=15) • Group 2: autologous FMT (n=6)	single FMT via endoscopy to distal duodenum	• 16S rRNA	• MRI-PDFF (allogenic#&: 17.52% (12.1-21.29%) vs 16.6% (12.26-24.8%); autologous#&: 30.38% (8.76-32.48%) vs 25.52% (8.06-32.89%)#&)	• HOMA-IR (allogenic#&: 3.88 (2.840-5.345) vs 3.52 (2.78-5.05); autologous#&: 4.88 (2.57-6.79) vs 4.96 (2.73-6.53)) • intestinal permeability (lactulose:mannitol urine test)	/	In group 1 compared to group 2: • (-) HOMA-IR • (-) MRI-PDFF • ↓small intestinal permeability

↑, significantly increased; ↓, significantly decreased; (-), not significantly altered.

*Oral probiotics containing a *Bifidobacterium* viable preparation and *Lactobacillus acidophilus* capsules; $ Valued as estimate [95%CI]; #Values before and after FMT; & Valued as median (IQR).

HOMA-IR: Homeostatic Model Assessment for Insulin Resistance; MASLD: metabolic dysfunction-associated steatotic liver disease; MRI-PDFF: magnetic resonance imaging-derived proton density fat fraction; NAFLD: nonalcoholic fatty liver disease; PAC, phenylacetic acid; PAG, phenylacetylglutamine; TARS, Threonyl-TRNA Synthetase 1; ZFP57, Zinc finger protein 57.

**Figure 1:**
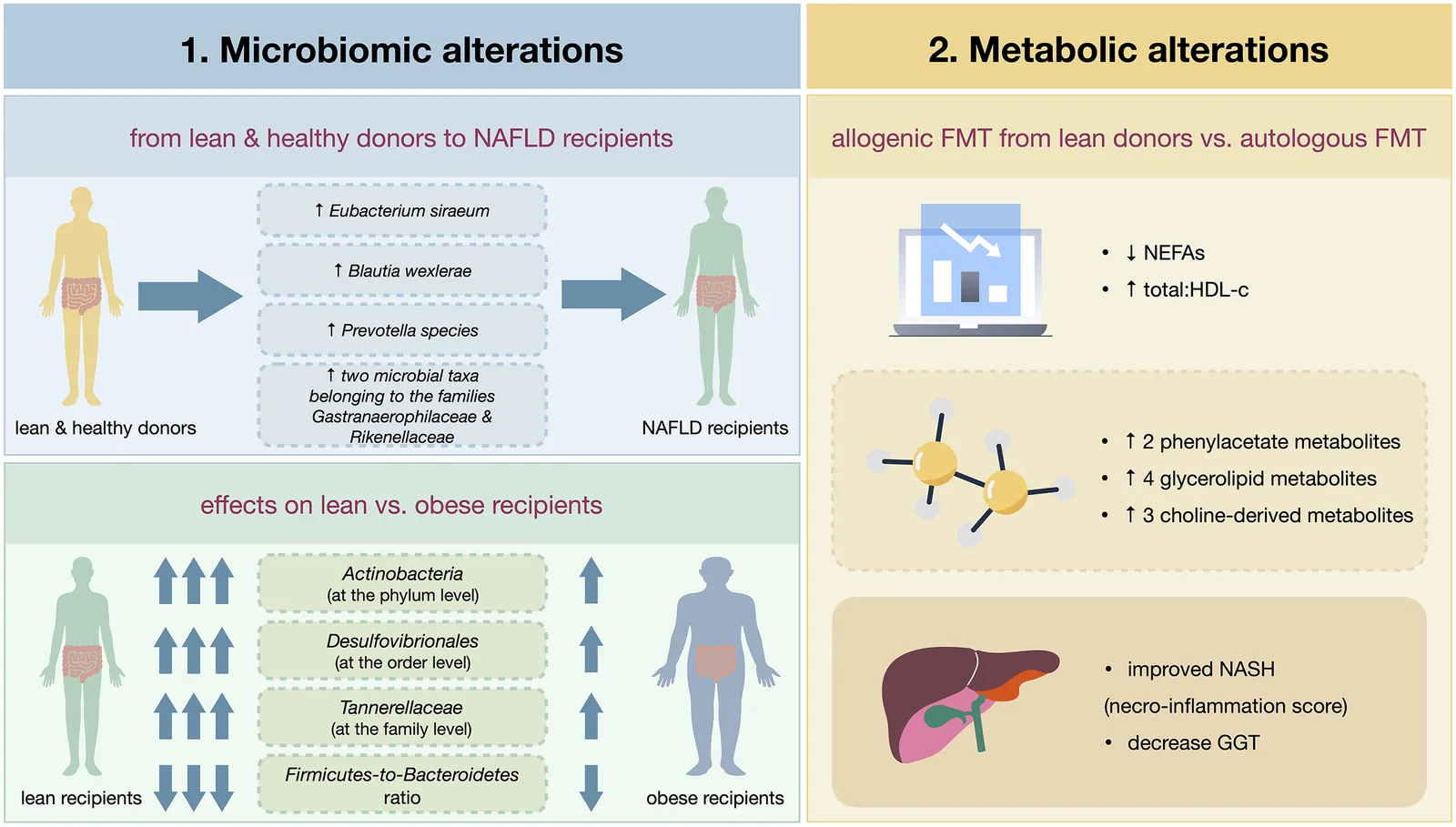
Benefits of FMT in patients with NAFLD. (1) microbiomic alterations: when the donors were lean and healthy, FMT increased the abundance of Eubacterium siraeum and Blautia wexlerae and Prevotella species, as well as two microbial taxa belonging to the families *Gastranaerophilaceae* and *Rikenellaceae* in NAFLD recipients. When the NAFLD recipients were lean, the abundance of *Actinobacteria* at the phylum level, the *Desulfovibrionales* at the order level and the *Tannerellaceae* at the family level were all significantly higher than that in obese patients after FMT, and the Firmicutes-to-Bacteroidetes ratio was significantly lower than that in obese patients after FMT. (2) metabolic alterations: when the FMT donors were lean, a reduced nonesterified fatty acids (NEFAs) and total:HDL cholesterol ratio, higher levels of two phenylacetate metabolites, as well as four glycerolipid metabolites and three choline-derived metabolites together with an improvement in NASH necro-inflammation score and decreased gamma-glutamyl transferase (GGT) were detected in allogenic FMT recipients than in autologous FMT recipients.

**Table 2:** Unpublished registered studies (clinicaltrials.gov) on the application of FMT in the treatment of patients with NAFLD.

Registration No.	Sponsor	Phase	Targeted disease	Source of material	Route of administration	Comparator	Outcome measures (primary)	Outcome measures (secondary)	Follow-up duration	Status (per registry)
NCT02469272	Lifespan (USA)	I	NASH	lean healthy donors	upper GI endoscopy	none	• degree of hepatic steatosis (MRI)	• Liver function tests • Markers of insulin sensitivity	12 weeks	recruiting
NCT03803540	Puerta de Hierro University Hospital (Spain)	I	NASH	lean healthy donors	upper GI endoscopy	none	• efficacy (historical diagnosis)	• safety • microbiota composition	72 weeks	not yet recruiting
NCT04014413	Chinese University of Hong Kong (China)	N/A	multiple diseases including NAFLD and NASH	not mentioned	not mentioned	none	• efficacy (number of improved patients)	• safety (treatment-related AEs)	1 year	recruiting
NCT04465032	Leiden University Medical Center (Netherlands)	IV	NAFLD	lean donor	upper GI endoscopy	own fecal transplantations	• liver fat accumulation (MRI)	• body weight, blood pressure, waist • β-cell function, insulin resistance • liver enzymes, bile composition • bacterial species • lipid homeostasis • psychological stress • physical activity	12 weeks	recruiting
NCT04594954	Institute of liver and biliary sciences (India)	N/A	obese NAFLD	not mentioned	upper GI endoscopy	diet + exercise without FMT	• proportion of patients achieving ≤5% of the weight loss (3 months)	• proportion of patients achieving ≤5% of the weight loss (6, 12 months) • metabolic-related measures	1 year	recruiting
NCT05622526	Instituto de Investigacion Marques de Vasldecilla (Spain)	II	NASH	MBK-01	oral capsules	placebo	• proportion of patients with improved fat fraction (MRI) • number of AEs, SAEs	• proportion of patients with improved lobular swelling and/or ballooning without worsening of fibrosis (MRI) • NAFLD Fibrosis score • body weight • blood pressure, lipid profile, carotid ultrasound • quality of life • patient-reported outcomes	72 weeks	not yet recruiting

### 3.1 Metabolic alterations

Following FMT, the average fat attenuation of lean NAFLD patients dropped to normal levels, while was barely changed in obese recipients (Xue et al., 2022). In addition, a beneficial lipid profile, including lower concentrations of non-esterified fatty acids (NEFAs), triglycerides and a decreased total to HDL cholesterol ratio, has been observed in recipients six weeks after allogenic FMT from lean donors compared to those receiving an autologous FMT with feces collected after diagnosis (Craven et al., 2020). Furthermore, two discriminative metabolites involved in phenylacetate metabolism (phenylacetylcarnitine and phenylacetylglutamine) were both significantly increased in allogenic FMT recipient but decreased in autologous FMT recipients, with feces collected after diagnosis, which was in association with the intestinal bacterial species *Firmicutes bacterium CAG 170* and *Gemminger formicilis* (Stols-Goncalves et al., 2023). Additionally, four discriminative metabolites related to glycerolipid metabolism (glycerol, 1-stearoyl-GPG (18:0), palmitoyl-arachidonoyl-glycerol (16:0/20:4) and 1-palmitoyl-GPG (16:0)) and three choline-derived metabolites (stearoylcholine, palmitoylcholine and oleoylcholine) were also identified to vary between these two groups of participants (Stols-Goncalves et al., 2023). Simultaneously, a trend towards improvement in histological NASH necro-inflammation score (an indicator of disease activity) and decrease gamma-glutamyl transferase (GGT) upon alterations in plasma levels of these metabolites was observed after autologous FMT with feces collected after diagnosis but not allogenic FMT (Witjes et al., 2020), indicating that allogenic FMT may induce changes in metaorganismal pathway from gut bacteria to liver.

### 3.2 Microbiomic alterations

Compared to healthy individuals, gut microbiota dysbiosis in NAFLD is characterized by reduced diversity of microorganisms and decreased beneficial microbiota, which may result in metabolic disorders and opportunistic pathogen infection. FMT from lean healthy donors may reverse this metabolomics signature directly. The consequence of microbiomic modulation includes alteration in NAFLD-associated hepatic DNA methylation. When FMT donors were lean and healthy, the abundance of *Eubacterium siraeum* and *Blautia wexlerae* was dramatically increased and hepatic DNA methylation profiles (mostly Threonyl-TRNA Synthetase 1 (TARS) as well as Zinc finger protein 57 (ZFP57)) were affected in recipients with NAFLD (Stols-Goncalves et al., 2023). These DNA methylation profiles may act as a hepatic regulator in response to gut-microbiota-derived signals (Stols-Goncalves et al., 2023). Similarly, allogenic FMT from lean donors has also been shown to affect the gut microbiome (particularly the introduction of *Prevotella* species) and mitochondrial-associated DNA methylome (Actin filament-associated protein 1 (AFAP)) of peripheral blood mononuclear cells in recipients with the metabolic syndrome by another clinical study (van der Vossen et al., 2021). Such a microbiome shift post FMT has been consistently observed in multiple animal-based studies and is often interpreted as a beneficial microbial signature for NAFLD (Zhou et al., 2017; Cho et al., 2018; Garcia-Lezana et al., 2018). Additionally, a persistent modification of the liver DNA methylation has also been identified in laboratory work that modulated gut microbiome in diet-induced NAFLD mice (Kim et al., 2019). Additionally, two microbial taxa belonging to the families *Gastranaerophilaceae* and *Rikenellaceae* were associated with triglyceride levels in MASLD patients after FMT from lean donors (Groenewegen et al., 2025).

When comparing lean NAFLD patients with obese ones, these lean patients have demonstrated a higher clinical efficacy following FMT intervention (Xue et al., 2022). This may be associated with a higher impact of FMT on the microbial community structure in lean individuals, since the abundance of *Actinobacteria* at the phylum level, the *Desulfovibrionales* at the order level and the *Tannerellaceae* at the family level were all significantly higher than that in obese individuals (Xue et al., 2022). Notably, those “harmful bacteria” that positively correlated with the inflammatory cascades and negatively correlated with body weight and blood glucose were more abundant in lean NAFLD patients (Delzenne and Bindels, 2018), whereas the “beneficial bacteria” that improve glucose and lipid metabolism and intestinal inflammation were less abundant in lean NAFLD patients (Wang et al., 2019a), which all contributed to the pathogenesis of fatty liver. Therefore, it is reasonable to conclude that lean NAFLD patients are more sensitive to microbiota-modulated treatment like FMT largely due to the nature of a more dominant role of imbalanced harmful/beneficial bacteria in the pathogenesis of the disease. In another study when observing the microbial diversity in obese patients, it was found that a markedly reduced microbial diversity and a disrupted *Firmicutes-to-Bacteroidetes* ratio in these obese NAFLD patients (Prasad Vudathaneni et al., 2025). An imbalanced *Firmicutes-to-Bacteroidetes* ratio (reduced *Bacteroidetes* and increased *Firmicutes*) has been associated with obesity via triggering the excessive calorie intake and abnormal lipid accumulation (Turnbaugh et al., 2006; Johnson et al., 2017). The increased relative abundance of *Firmicutes* may promote energy harvesting from indigestible polysaccharides, enhancing lipogenesis and hepatic fat accumulation (Xie and Halegoua-DeMarzio, 2019). Thus, such an imbalanced *Firmicutes-to-Bacteroidetes* ratio may also contribute to the onset of NAFLD.

## 4 Impact of specific post-FMT microbiome shifts on the gut of NAFLD patients

As mentioned above, FMT affected the gut microbiome of NAFLD patients by increasing the abundance of *Eubacterium siraeum*, *Blautia wexlerae*, as well as *Prevotella* species. The abundance of *Eubacterium siraeum* was previously shown positively correlated with high-density lipoprotein-cholesterol (HDL-c) levels in non-human primate models of NAFLD (Jiao et al., 2021; Newman et al., 2021). Meanwhile, the upregulation of *Blautia wexlerae* has been observed in NAFLD patients administered a hypocaloric hyperproteic diet, indicating the potential probiotic property of this anaerobic bacterium for NAFLD (Pataky et al., 2016).

The alterations in gut barrier integrity and gut permeability is another contributing factor for determining which and how much the microbiota-derived products may reach the liver via the activation of pathogen-associated molecular patterns (PAMPs) and damage-associated molecular patterns (DAMPs), and their downstream effects on the lipid metabolism, inflammatory responses and fibrosis of the liver (Leung et al., 2016; Witjes et al., 2020; Sharpton et al., 2021; Tilg et al., 2021a). The imbalance of microbiota, which can affect the content of tight junction proteins (ZO-1, occluding, claudin-1), is one of the main causes of impaired intestinal barrier function (Li et al., 2025). Upon the increase in intestinal permeability, more lipopolysaccharides (LPS) from Gram-negative bacteria are absorbed into the blood through the intestine, thus increases serum LPS levels, which bind to toll-like receptors (TLRs) in the liver and aggravate fatty liver through the LPS/TLR4/NF-κB pathway (Xue et al., 2023; Buchynskyi et al., 2025). In response to LPS, Kupffer cells, the resident liver macrophages, and hepatic stellate cells are activated and then release pro-inflammatory cytokines, which further elevates insulin resistance and promotes hepatic inflammation and fibrosis, thus having a central role in MASLD progression (Dua et al., 2025). It is noteworthy that a significant improvement in small intestinal permeability, which was assessed using the lactulose: mannitol urine test, was observed in NAFLD patients receiving allogenic, but not autologous FMT with feces collected after diagnosis (Craven et al., 2020). This could be at least partially explained by the post-FMT accumulation of butyrate-producing bacteria including Bacteroidetes and rumen bacteria (Poeker et al., 2018), leading to a protection of intestinal mucosal barrier via an increased production of butyric acid (Wang et al., 2012). In addition, there have been positive relations between the abundance of *Firmicutes* (Cogger et al., 2016) or *Eubacterium hallii* (Witjes et al., 2020) and liver endothelial cell integrity and function.

## 5 FMT-derived benefits in metabolic pathways

It was noted that FMT exerted beneficial effects on NAFLD through some potential mechanisms by improving hepatic insulin sensitivity, promoting the production of short-chain fatty acids (SCFAs), affecting lipogenesis in the liver, as well as bile acid remodeling (**Figure 2**).

**Figure 2:**
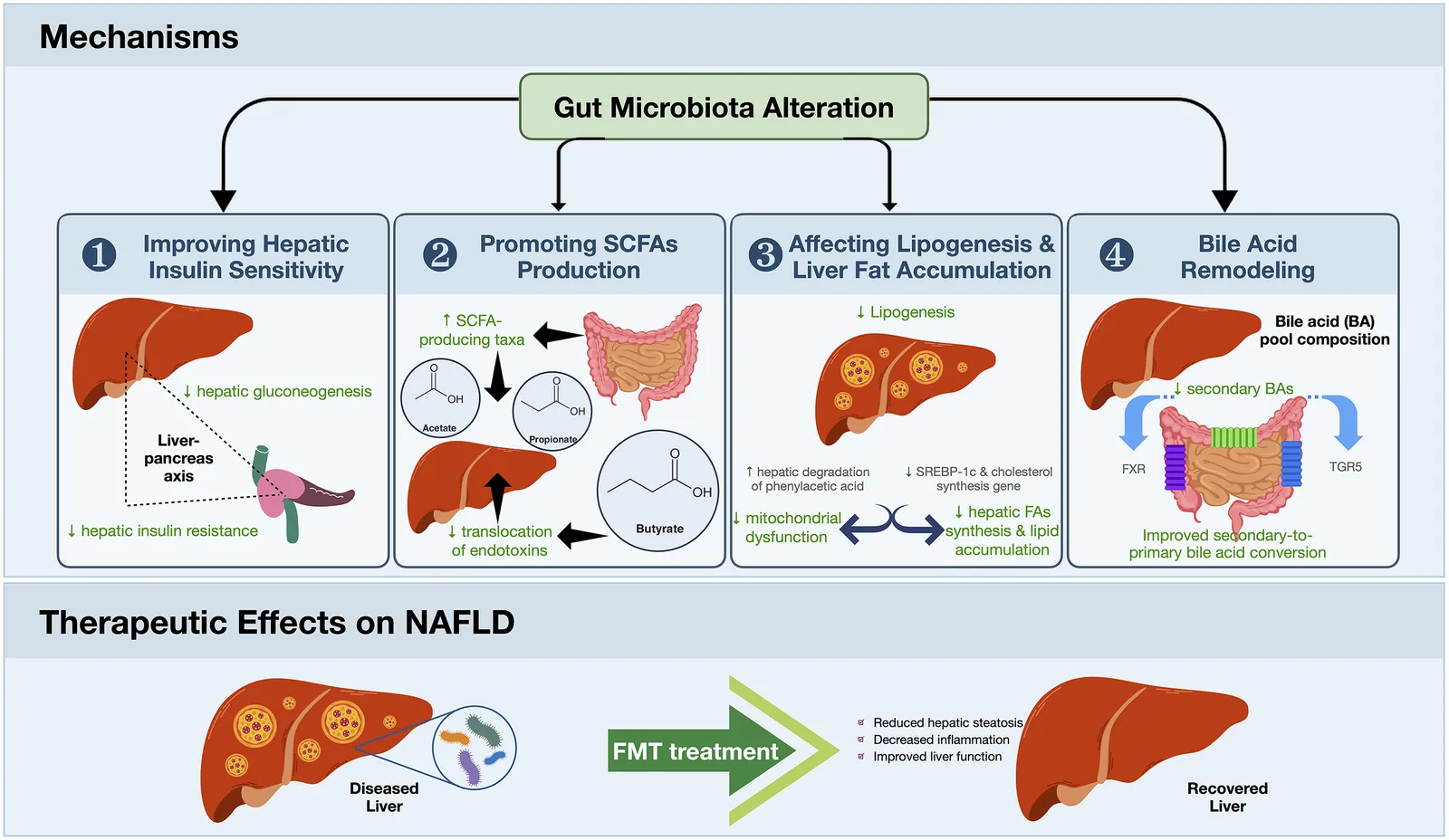
FMT-derived benefits in metabolic pathways. (1) Improving hepatic insulin sensitivity: the hepatic insulin resistance was alleviated along with a reduced hepatic gluconeogenesis via the modulation of liver-pancreas axis; (2) Promoting short-chain fatty acids (SCFAs) production: the supplementary of SCFA-producing taxa was reported to reduce the translocation of insulin-resistance-associated endotoxins into the bloodstream and prevent the harmful downstream effects on the liver; (3) Affecting lipogenesis and liver fat accumulation: an increased hepatic degradation of phenylacetic acid post FMT treatment reduced mitochondrial dysfunction, and the produced SCFAs resulted in an enhancement of leptin signaling for the suppression of expression of SREBP-1c and cholesterol synthesis gene, thereby reducing hepatic lipid accumulation; (4) Bile acid remodeling: the FMT-modified gut dysbiosis shifts the bile acid pool back towards its normal function, thus reducing the interactions between secondary BAs with the farnesoid X receptor (FXR) and the Takeda G-protein-coupled receptor 5 (TGR5).

### 5.1 Hepatic insulin sensitivity

As a result of altered circulating amino acids (especially branched-chain amino acids (BCAAs)) and impaired amino acids metabolism affected by gut microbiome (Newgard et al., 2009; Pedersen et al., 2016), the insulin resistance is regarded as a central metabolic driver of NAFLD progression, which drives increased delivery of hepatic free fatty acids, promotes hepatic lipogenesis, lipotoxic milieu and triggers NASH (Tilg et al., 2021b). Specifically, the decreased plasma level of phenylalanine has been associated with early hepatopathy (Ishii et al., 2003). Phenylalanine is converted into tyrosine in the liver under normal circumstances, however, when the liver loses its capacity in metabolizing this BCAA, it results in a negative association between phenylacetylglutamine and NAFLD (Hoyles et al., 2018), whereas a positive association between phenyllactic acid and NAFLD (Caussy et al., 2018). The effect of FMT from lean to obese individuals with metabolic syndrome on the improvement of hepatic insulin sensitivity have been discovered in multiple clinical studies (Vrieze et al., 2012; Kootte et al., 2017; Yu et al., 2020).

As two important risk factors for the development of NASH, both insulin resistance and obesity seemed to aggravate the progression of portal hypertension (Berzigotti et al., 2017). Heightened portal pressure was not uncommon even for early-stage NAFLD (Mendes et al., 2012). A previous animal study has pointed out the critical role of intestinal microbiota in the pathogenesis of portal hypertension in NASH, triggering hepatic insulin resistance and endothelial dysfunction within a short period of time (8 weeks) (Garcia-Lezana et al., 2018), indicating the potential of microbiome-based therapeutic strategy targeting portal hypertension in NASH.

### 5.2 SCFA production

Short-chain fatty acids (SCFAs) are metabolites of indigestible carbohydrates produced by a variety of microbiota in the large intestine and are capable of inhibiting cholesterol synthesis, obesity, and inflammation (Hao et al., 2020). This is because SCFAs inhibit the activation of nuclear factor-kappa B (NF-κB) by binding to free fatty acid G-protein-coupled receptors FFA2 and FFA3, thereby, suppressing the release of tumor necrosis factor-ɑ (TNF-ɑ) and interleukin-6 (IL-6) (Alvarez-Curto and Milligan, 2016). Chronic inflammation may aggravate insulin resistance in insulin-sensitive organs including the liver, and lead to impaired glucose tolerance (Okamura et al., 2022). Via the recently proposed ‘gut-liver axis’, the liver may be more easily exposed to gut microbiota-derived metabolic products including the SCFAs (Tilg et al., 2021a). Butyrate is a SCFA produced by the breakdown of fiber by a wide range of gut microbiota, which increases the intestinal barrier integrity and reduces the translocation of LPS. A decreased level of butyrate has been verified in animal model of NAFLD and insulin resistance (Cani et al., 2007), hence, the supplementation of butyrate producing taxa has been attempted in the treatment of these conditions. A study by Vrieze et al. showed that FMT from lean donors transferred by single administration via duodenal tube into obese participants increased insulin sensitivity via upregulation of potent butyrate producers *Roseburia intestinalis* and *Eubacterium hallii* (Vrieze et al., 2012). *Eubacterium hallii* has been found to use lactic acid for butyrate production and exert metabolic benefits to humans (Gilijamse et al., 2020). Consequently, the increased butyrate reduced the translocation of insulin-resistance-associated endotoxins into the bloodstream (Vrieze et al., 2012). Therefore, the engraftment of the SCFA-producing and anti-inflammatory taxa constitutes the foundational ecological environment for downstream effects on the liver.

### 5.3 Lipogenesis

Although animal studies have demonstrated attenuation of liver fat following FMT treatment (Zhou et al., 2017; Burz et al., 2021), contradictory results have been reported in clinical trials. The elevated plasma levels of the metabolites, phenylacetylglutamine (PAG) and phenylacetylcarnitine (PAC) in NAFLD patients receiving allogenic FMT suggested an increased hepatic degradation of phenylacetic acid, subsequently reducing phenylacetic-acid-induced mitochondrial dysfunction and hepatocyte lipid accumulation (Caussy and Loomba, 2018). A slight reduction in liver fat was also observed by fibroscan 4 weeks after FMT via colonoscopy and enema in another clinical study (Xue et al., 2022). However, no significant decrease in liver fat, or MASLD severity scores in liver biopsies or magnetic resonance imaging-derived proton density fat fraction (MRI-PDFF) was detected when comparing allogeneic versus autologous FMT with feces collected after diagnosis in three smaller-scaled RCT recruiting MASLD patients (Craven et al., 2020; Witjes et al., 2020; Groenewegen et al., 2025).

SCFAs such as butyrate and propionate could activate GPR43 receptor and inhibit histone deacetylases (HDACs) in hepatocytes (Li et al., 2024), subsequently upregulating PPAR-associated fatty acid oxidation, and enhancing leptin signaling for the suppression of expression of SREBP-1c (the master transcription factor driving hepatic fatty acid synthesis) and cholesterol synthesis gene, thereby reducing hepatic lipid accumulation (Wang et al., 2025).

### 5.4 Bile acid remodeling

In NAFLD, the disrupted secondary-to-primary bile acid conversion is driven by gut dysbiosis. This disrupted bile acid pool caused some direct consequences on the liver because bile acids are potent signalling molecules. For instance, the secondary bile acids interact with the farnesoid X receptor (FXR) and the Takeda G-protein-coupled receptor 5 (TGR5), exerting influence over hepatic lipid homeostasis (Buchynskyi et al., 2025). On one hand, selective inhibition of intestinal FXR has been shown to improve insulin resistance and NAFLD in mice, possibly via the metabolism of bile acids (Jiang et al., 2015). On the other hand, TGR5 activation in intestinal L cells stimulates GLP-1 secretion and promotes mitochondrial biogenesis and anti-inflammatory signalling in the liver. When FMT engrafts bile acid-modifying bacteria from the donor, it shifts the pool back towards its normal function (Buchynskyi et al., 2025; Dua et al., 2025).

## 6 A quantified successful engraftment does not promise clinical benefits

The determination of microbiota composition and engraftment required stool samples from both recipients and donors. Species engraftment fraction (SEF) measures the proportion of donor species present in patients post-FMT, excluding those already present in patients pre-FMT (van Lingen et al., 2024). However, donor microbiota engraftment appeared donor-specific but not response-specific, which means even with the detection of the engraftment of the donor microbiota, clinical benefits in liver fat content, lipid profiles, liver biochemistry or glucose tolerance for MASLD patients could still be lacking (Groenewegen et al., 2025).

Based on the understanding of the critical role of gut microbiota in the pathogenesis of NAFLD, it was not difficult to understand the clinical benefits which may be expected following the FMT intervention (**Table 3**). However, the association between microbiome shifts and insulin sensitivity have been varied in different studies. Groenewegen et al (Groenewegen et al., 2025). reported that microbiota diversity showed no significant association with continuous changes in the Homeostasis Model Assessment of Insulin Resistance (HOMA-IR) or serum triglycerides and an even stronger negative association with the MRI-PDFF, which was used as an indicator for hepatic steatosis, in the autologous FMT group with feces collected after diagnosis, as compared to the allogeneic FMT group. Specifically, the genus *Stercorousia* (belonging to the *Gastranaerophilaceae* family) showed a stronger negative association with triglycerides in the allogeneic FMT group compared to the autologous FMT group, whereas *Alistipes putredinis* (belonging to the *Rikenellaceae* family) showed a weaker positive association, which may potentially explain the mitigation of serum triglyceride levels (Groenewegen et al., 2025). The biosynthetic gene clusters linked to *Stercorousia* have been negatively associated with BMI (Wang et al., 2019b), whereas the *Alistipes putredinis* has been negatively associated with MASLD, and was linked to enhanced weight loss from physical activity (Yang et al., 2023; Schwenger et al., 2024).

**Table 3:** Main outcomes measured in FMT-NAFLD trials.

Category	Measurement	Method	Timepoint of measurement post FMT	Reference values (if applicable)
Liver measures	Hepatic PDFF	MRI	2 weeks, 6 weeks, 6 months	A relative PDFF reduction of ≥30% related to an intervention predicts reduction of the NAS by ≥2 points (Panagiotopoulos et al., 2026)
	FibroScan-based severity grading	Liver elastography	1 month	Cutoff of FAST (FibroScan AST) was 0.35 for sensitivity of ≥0.90 and 0.67 for specificity ≥0.90 in suspected NAFLD (Newsome et al., 2020)
	NAS, necro-inflammation score, fibrosis score, steatosis score	liver biopsy and histopathology	24 weeks	A ≥2 points improvement in NAS was applied as a method for evaluating treatment response for NASH (Tamaki et al., 2021)
	Hepatic gene expression	RNA-sequencing analysis	24 weeks	/
Microorganism measures	gut microbiota composition	16S rRNA	24 weeks	/
Metabolic measures	HOMA-IR	formulated calculator	2 weeks, 6 weeks, 6 months	The optimal HOMA-IR cut-off for NAFLD was 1.9 (sensitivity 87%, specificity 79%) (Isokuortti et al., 2017)
	glucose tolerance	glucometer-based or laboratory-based	12 weeks	/
Intestinal permeability	lactulose:mannitol urine test	LC-MSMS	2 weeks, 6 weeks, 6 months	/

HOMA-IR, Homeostatic Model Assessment for Insulin Resistance; NAS, NAFLD activity score; PDFF, proton density fat fraction.

A critical mechanistic nuance is that the NAFLD hepatic environment itself impairs FMT efficacy through feedback effects on gut ecology (**Figure 3**). Elevated bile acid concentrations refluxing into the intestine, systemic hyperglycaemia, and heightened portal pressure all modify the luminal environment in ways that reduce engraftment success of donor species. The healthy gut-liver axis involves a stable gut barrier, regulated immune responses, and microbial production of SCFAs, gut mucosal hormones such as FGF 15/19 and bile acid metabolism, which together support liver homeostasis (Tilg et al., 2022). In contrast, dysbiosis in NAFLD/NASH leads to barrier dysfunction, increased intestinal permeability, translocation of microbial products including LPS, decreased SCFA and bile acid production, immune imbalance, and activation of pro-inflammatory pathways, ultimately promoting hepatic steatosis, inflammation, and fibrosis (Bahitham et al., 2025). This may explain the reason why NAFLD is so difficult to reverse with a single FMT intervention and why patients with higher baseline microbiome diversity (less severe dysbiosis) tend to respond less dramatically to FMT. For instance, some short-chain fatty acids produced by gut microbiota such as propionate and acetate were able to reach liver and exert dual effects in NAFLD (Hasain et al., 2020; Tsai et al., 2021). Therefore, successful colonization may increase SCFA-producing microbiota but not necessarily exert beneficial effects. The secondary bile acids are FXR and TGR5 ligands that are converted from primary bile acids by gut microbiota (Sharpton et al., 2021; Li et al., 2023). Though bile acid metabolism may be recovered, such process involves contribution from specific strains as well as sufficient liver function. A successful engraftment thus does not guarantee sufficient effects. In addition, NAFLD is featured by systematic inflammation (Luo et al., 2017), which potentially affects growth of donor microbiota despite initial success of engraftment. Therefore, FMT may not exert desired effects to NAFLD despite successful colonization, which requires further investigation.

**Figure 3:**
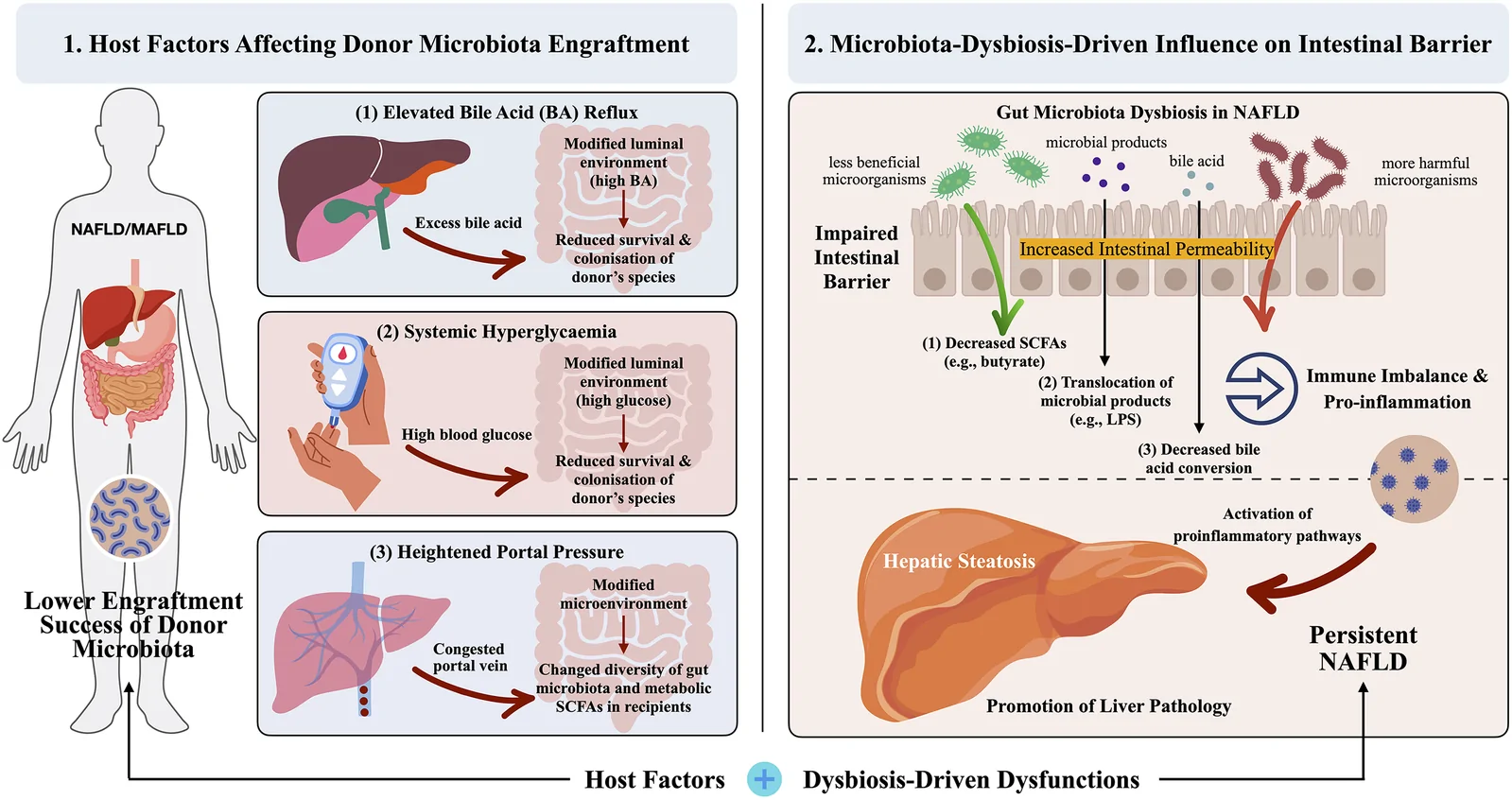
Host factors and dysbiosis-driven dysfunctions responsible for FMT-resistant NAFLD. (Left): Host factors affecting donor microbiota engraftment. Elevated bile acid reflux into the intestine, systemic hyperglycemia and heightened portal pressure all modified the luminal environment and reduced the engraftment of donor’s species. (Right): Dysbiosis in NAFLD leads to impaired intestinal barrier, which increased the intestinal permeability, subsequently affecting the production of SCFAs, translocation of microbial products and conversion of bile acids. The immune imbalance and the activation of pro-inflammatory pathways driven by these events ultimately promote pathogenesis in the liver.

## 7 Limitations and future perspectives

### 7.1 Small sample size

The most fundamental problem questioning the FMT-NAFLD evidence is the small sample sizes, making these underpowered studies more as conceptual works. Among the limited number of clinical studies that have assessed the role of FMT in MASLD, their sample sizes were grossly underpowered to detect meaningful changes in primary hepatic endpoints, particularly fibrosis regression, and FMT may only play a minimal role in the complex and confounded MASLD pathological background (Saha and Schnabl, 2025), making it impossible to reliably identify microbiome signatures associated with FMT responsiveness. As a consequence, it requires large cohorts and long observation periods to capture these histological signals.

### 7.2 Insufficient follow-up duration

The natural history of NAFLD evolves over years to decades, yet virtually all FMT trials in this context report outcomes at 12–24 weeks. This is mechanistically problematic for the following reason. Hepatic fibrosis and even steatohepatitis requires substantially longer periods to show histological change; and secondly, the ecological re-establishment of a stable post-FMT microbiome community takes months, and its downstream metabolic effects on the liver operate through slower transcriptional and epigenetic remodelling rather than rapid biochemical shifts. For instance, FMT recipients with metabolic syndrome have been followed up for four years for observing effects on the improvements in body composition and metabolic health, including smaller waist circumference, lower total body fat, reduced metabolic syndrome severity score, and reduced systemic inflammation, which were not apparent at shorter follow-up timepoints (Wilson et al., 2025). Therefore, the failure of current NAFLD trials to demonstrate post-FMT clinical benefits may reflect temporal mismatch rather than genuine biological ineffectiveness.

### 7.3 Lack of histological primary endpoints

Liver biopsy is so far the reference standard for the diagnosis of NAFLD (Rinella et al., 2019), however, it was performed very occasionally in FMT-NAFLD studies (Stols-Goncalves et al., 2023). Furthermore, no study to date has found a change in clinically meaningful outcomes such as steatosis or fibrosis grade in NAFLD (Saha and Schnabl, 2025), yet virtually none of the completed trials were adequately powered or designed with histology as a primary outcome. Most trials have relied on non-invasive endpoints for hepatic steatosis such as imaging (MRI-PDFF, FibroScan) and serum liver enzymes, whose correlation with histological response in interventional trials is imperfect. Therefore, this research field urgently needs adequately powered RCTs with paired liver biopsies as one of the primary endpoints.

### 7.4 Lack of a standardized FMT protocol for this patient cohort

The methodology of donor screening and characterization, the number of FMT doses (single vs. three consecutive vs. six weekly), route of administration (colonoscopy, nasoduodenal tube, oral capsules) (**Table 4**), approaches of preparations (fresh vs. frozen preparations, use vs. non-use of antibiotic pre-conditioning) have all met inconsistency in different studies, which may thereby yield an insignificant or negative clinical outcome in liver biochemistry, glucose tolerance and HOMA-IR (Groenewegen et al., 2025). This heterogeneity impeded cross-trial comparisons, thus, a standardized FMT protocol is urgently needed for patients with NAFLD.

**Table 4:** Comparing different FMT delivery routes.

Items for comparison	Nasogastric/nasoduodenal/nasojejunal tube	Esophagogastro-duodenoscopy (EGD)	Oral capsules	Colonoscopy	Retention enema
Delivery site	stomach, duodenum, or jejunum	stomach or duodenum	small intestine and/or colon	cecum, terminal ileum, distal colon	Distal colon (rectosigmoid)
Procedure complexity	Low; bedside placement	High; endoscopy suite	Very low; self-administered	High; endoscopy suite	Low; clinic or at-home
Outpatient/at-home use	Limited (in-clinic placement)	No (endoscopy suite required)	Excellent for maintenance dosing	No (endoscopy suite required)	Good (self-administration possible)
Bowel preparation	No	No standard prep	No	Yes, PEG-based prep	optional, minimal
Volume per session	up to 500mL (2-3min/50mL) (Cammarota et al., 2017)	up to 500mL (Cammarota et al., 2017)	15–40 capsules (Kamath et al., 2025)	200-500mL fecal suspension (Cammarota et al., 2017)	up to 450mL (Skjevling et al., 2023)
Stool mass equivalent	~30-50g (Gong et al., 2025)	~30-50g (Gong et al., 2025)	~25-30g (Kamath et al., 2025)	~20-100g feces (Cammarota et al., 2017)	~50g (Skjevling et al., 2023)
Application in metabolic indications	Singe nasoduodenal dose used in metabolic syndrome (Vrieze et al., 2012)	Used in steatohepatitis (Witjes et al., 2020), NAFLD (Craven et al., 2020), MASLD (Groenewegen et al., 2025)	Used in obesity (Allegretti et al., 2020; Yu et al., 2020)	Used in NAFLD with combined route (enema) administration (Xue et al., 2022)	Used in metabolic syndrome (Piwchan et al., 2025)
Common minor AEs	nausea, cramping, bloating, sore throat, regurgitation	sore throat, bloating, sedation-related AEs (if used)	mild GI symptoms, bloating	bloating, cramping, transient diarrhea	cramping, urgency, incomplete retention
Key limitations	Tube burden, aspiration risk	Cost, sedation, impractical for repeat	Capsule burden	Cost, sedation, bowel prep burden, impractical for repeat	Limited proximal reach, variable retention

AEs, adverse events; PEG, polyethylene glycol.

### 7.5 Lack of a stratification and exclusion criterion of enrolled cohorts

The majority of existing trials have enrolled patients with NAFLD as a broad category without stratifying by liver disease stages (simple steatosis vs. NASH vs. early fibrosis, according to assessments such as the necro-inflammation score), or BMI phenotypes (lean vs. overweight vs. obese), or ruling out metabolic baseline comorbidities (e.g., presence/absence of T2DM, severity of insulin resistance), or baseline interventions (e.g., dietary intervention and lifestyle modification), or concomitant medications that may alter the gut microbial compositions (e.g., proton pump inhibitors and antibiotics) (Maier et al., 2018). This is scientifically unjustifiable given that the gut microbiome signatures, portal endotoxin flux, and intestinal barrier function differ profoundly across these disease stages and phenotypes. FMT has demonstrated clinical efficacy in NAFLD with lean NAFLD patients responding better than obese NAFLD patients, yet the use of such distinction as an enrolment stratification criterion is important (Cao et al., 2025). In addition, heterogeneities of sex and ethnicity were present across existing studies. Specifically, the proportion of males ranged from 29% (Craven et al., 2020) to 91% (Witjes et al., 2020), and the countries where these studies were performed covered Europe, North America and Asia. Furthermore, the performance of current biomarker showed heterogeneity across age groups. The heterogeneities in these crucial demographic features and study parameters cause challenges for synthesization of different studies and therefore, yielding validation by studies with more homogeneous features in the future.

## 8 Conclusion

The research on utilizing FMT in NAFLD patients is still scarce, and the correlation between ‘a successful transplantation’ and ‘beneficial clinical outcomes’ is not yet fully understood. Evidence from multiple studies on NAFLD have all proved that these patients were more sensitive to FMT in both the microbiomic and metabolic aspects compared to their comparators in the obese group, possibly due to the benefits derived from the successful engraftment of donor’s microbiota post FMT. However, it has been observed from the clinical practice that an identified successful engraftment is not a promise of clinical efficacy in NAFLD patients, which could at least partially be explained by host factors such as elevated bile acid concentrations refluxing into the intestine, systemic hyperglycaemia, and heightened portal pressure that all modify the luminal environment in ways that reduce engraftment success of donor species. Limitations in current studies include the underpowered sample sizes, insufficient follow-up durations, and lack of historical primary endpoints, standardized FMT protocol and stratification and exclusion criterion for patients with NAFLD (**Figure 4**). Therefore, as a potential therapeutic strategy for NAFLD, the application of FMT in the treatment of NAFLD warrants further investigations and clinical verifications.

**Figure 4:**
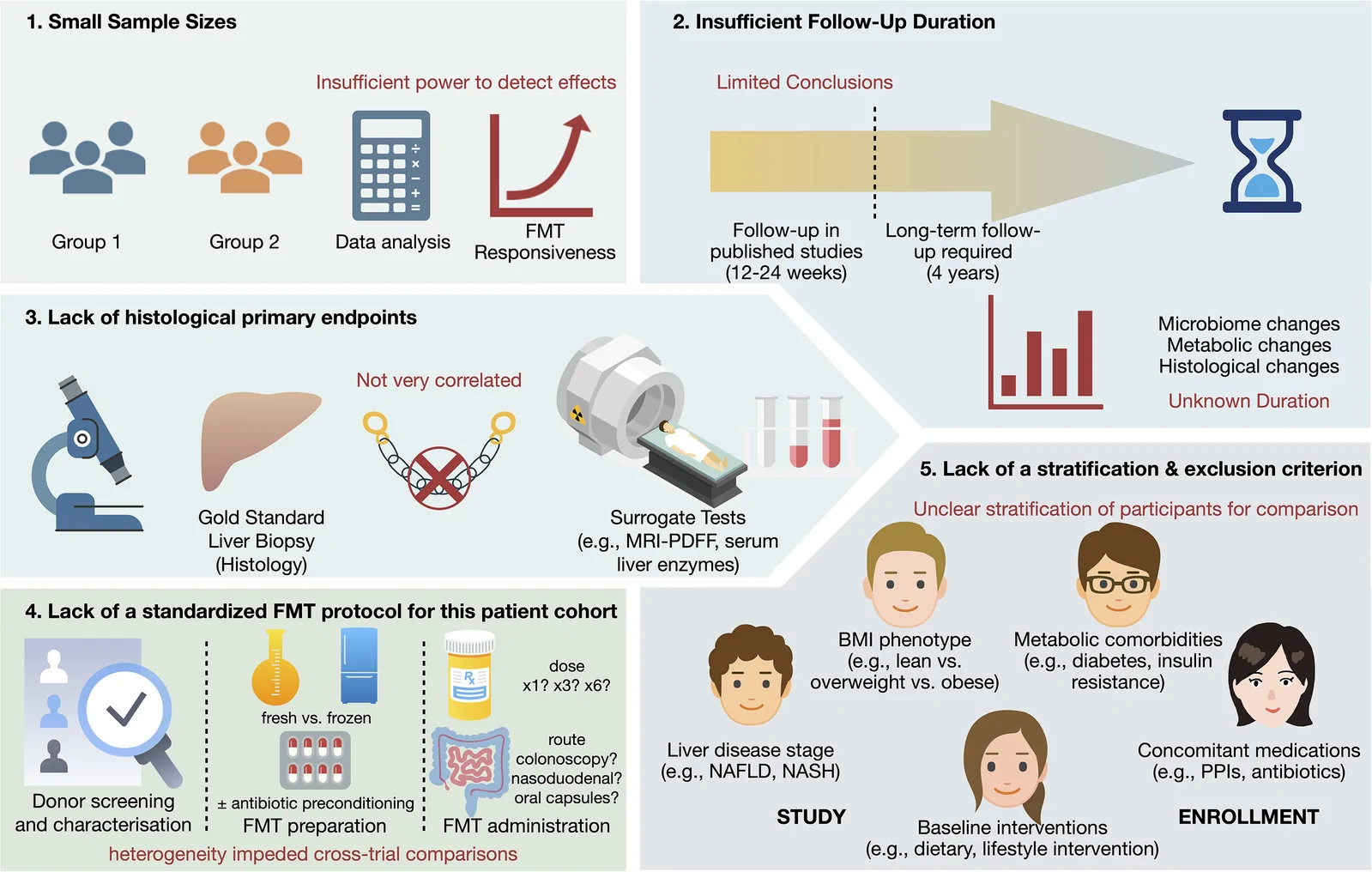
Limitations in current FMT-NAFLD research. (1) small sample size, which were grossly underpowered to detect hepatic endpoints and measure FMT responsiveness. (2) insufficient follow-up duration, which was problematic for following up changes in microbiome community and its downstream metabolic effects. (3) Lack of histological primary endpoints from liver biopsy, which was replaced by imaging and serum liver enzymes but imperfectly correlated with the gold standard. (4) Lack of a standardized FMT protocol including donor screening and characterization, the dosage and route of FMT administration, approaches of preparations all impede the comparison across different studies. (5) Lack of a stratification and exclusion criterion of enrolled recipients according to individual’s liver disease stages, or BMI phenotypes, or ruling out metabolic baseline comorbidities, or baseline interventions, or concomitant medications that may alter the gut microbial composition.
